# Infection caused by a cryptic fungal species, Blastomyces gilchristii, in a tiger

**DOI:** 10.1099/acmi.0.001011.v3

**Published:** 2025-08-19

**Authors:** Sreekumari Rajeev, Porsha Reed, Alejandro Llanes, Rebekah Jones, Andrew Cushing, Linden E. Craig, Brian Johnson

**Affiliations:** 1 Department of Biomedical and Diagnostic Sciences, College of Veterinary Medicine, Knoxville, TN, USA; 2 Centro de Biología Celular y Molecular de Enfermedades, Instituto de Investigaciones Científicas y Servicios de Alta Tecnología (INDICASAT AIP), Panama City, Panama; 3 Department of Small Animal Clinical Sciences, College of Veterinary Medicine, University of Tennessee, Knoxville, Tennessee, USA

**Keywords:** *Blastomyces gilchristii*, cryptic fungus, tiger

## Abstract

Blastomycosis is a serious fungal disease affecting humans and animals. It is typically caused by the thermally dimorphic fungus, *Blastomyces dermatitidis*. In this report, we describe an infection caused by the cryptic fungal species, *Blastomyces gilchristii*, in a tiger (*Panthera tigris*).

## Data Summary

All relevant data used in this study are presented within the manuscript. The DNA sequences from the isolate recovered in this study are available through BioProject PRJNA767594 (*SRR16127079*).

## Introduction

Blastomycosis is a serious fungal disease of humans and animals primarily caused by the thermally dimorphic fungal species *Blastomyces dermatitidis* [1]. While most prevalent in North America (endemic in the Ohio and Mississippi River valleys and around the Great Lakes), its geographic distribution is expanding globally and exhibiting dynamic changes [2]. Most infections are asymptomatic, but in some hosts, infections can result in severe respiratory infections, skin lesions and multisystemic manifestations [1]. The disease is very similar to other dimorphic fungal infections caused by *Histoplasma* and *Coccidioidomyces*. The diagnosis of blastomycosis typically involves identifying unique morphological fungal features using cytology, histopathology and culture. A commercial enzyme immunoassay (MiraVista Diagnostics, Indianapolis, IN) that detects the fungal cell wall component galactomannan in urine or serum is a useful noninvasive assay for the diagnosis of blastomycosis, and the cross-reactivity with *Histoplasma *is common [1]. PCR-based methods exist, but their availability is currently limited to specialized reference laboratories. Amphotericin B or the azole group of drugs is commonly used for treating blastomycosis. Blastomycosis in the USA is only reportable in a few endemic states, so there is a lack of information from other areas [2]. Although blastomycosis was assumed to be caused by a single species, *B. dermatitidis*, with recent advances in sequencing, scientists have identified other *Blastomyces* species that can cause infections [3,4]. In this report, we describe an infection caused by a cryptic fungal species, *Blastomyces gilchristii*, in a tiger (*Panthera tigris*).

## Case report

### Clinical case

An adult female tiger was presented to the University of Tennessee College of Veterinary Medicine teaching hospital with an acute onset of neurological signs. This tiger was born in Tennessee, and no travel history was reported. The tiger was seen previously at the University of Tennessee College of Veterinary Medicine in March 2009 for suspected bite wounds and in April 2011 for a laparoscopic ovariectomy. A complete blood count and chemistry and urine analysis were unremarkable except for the presence of white blood cells in the urine. The patient was treated with cefovecin and fluconazole empirically, with no improvement, and the patient’s condition quickly deteriorated and was euthanized.

### Necropsy and histopathology findings

**Fig. 1.**
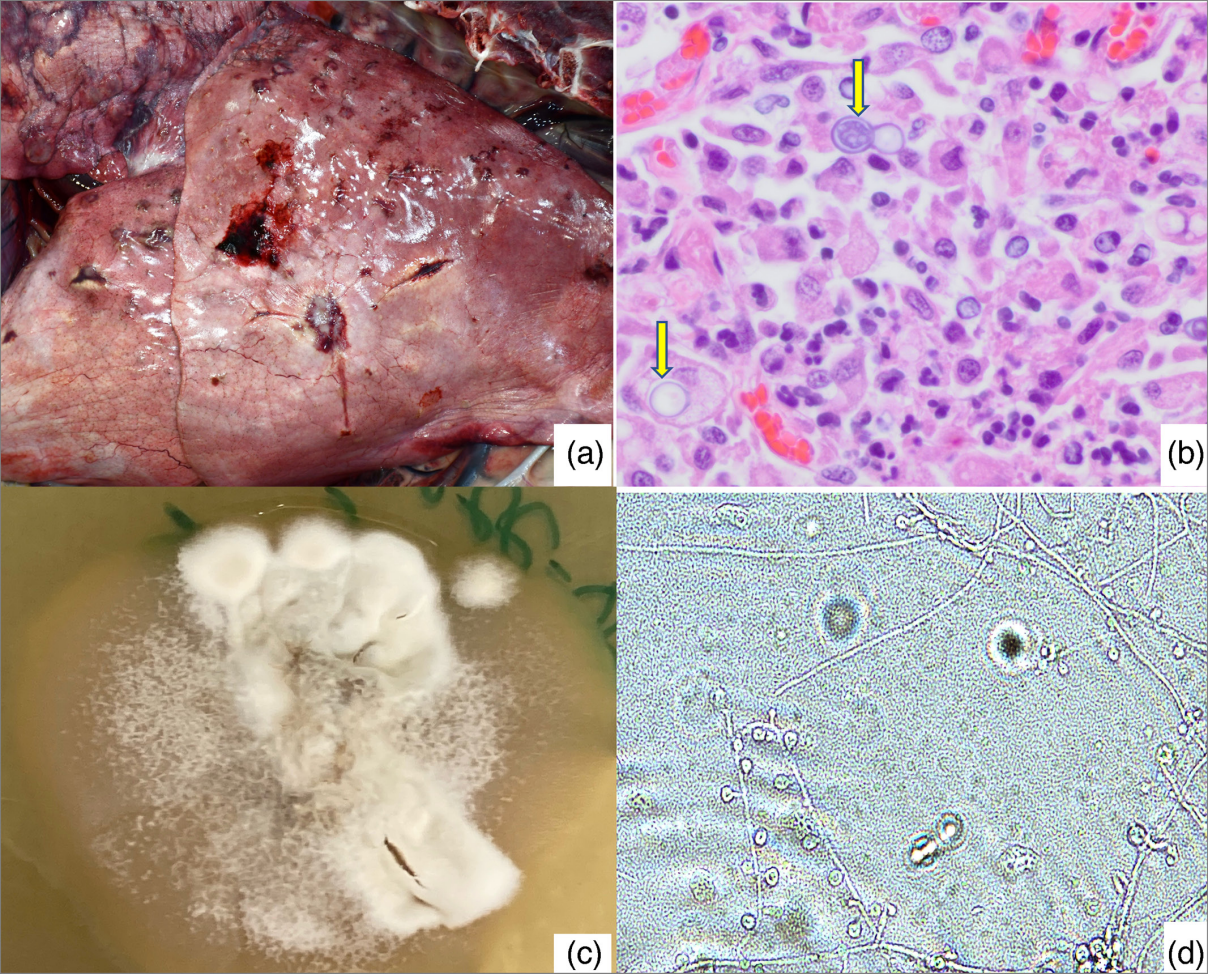
(a) Lung with multifocal nodules; (b) H&E-stained images of lung histopathology (600× magnification) showing yeast cells (yellow arrows); (c) fungal colony in the mycelial form growing at 30 °C in inhibitory mould agar; (d) lactophenol aniline blue dye-stained fungal colony; see the lollipop-shaped microconidia arising from the hyphae (400× magnification).

Major necropsy findings included multifocal nodules in the lungs (Fig. 1a), moderate acute multifocal suppurative dermatitis and moderate regionally extensive subcutaneous oedema. In addition, multifocal thyroid follicular cysts and adenomas were observed. On histopathology, areas of the cerebrum were expanded and replaced by macrophages, neutrophils, plasma cells, cellular debris and intrahistiocytic and free 15–25 µm yeasts with thick double-contoured walls and broad-based budding with occasional basophilic nuclear material. Similarly, within the nodules, the lung parenchyma was effaced by inflammation and yeast cells as described above (Fig. 1b). The final diagnosis was disseminated mycosis with encephalitis and pneumonia.

### Microbiology

Lung tissue culture on Inhibitory Mould Agar at 30 °C yielded tan, glabrous fungal colonies with a prickly centre after 2 weeks of incubation. Microscopic examination after lactophenol aniline blue staining revealed septate hyphae with variable-sized lollipop-like conidia. Yeast-phase conversion at 35 °C required multiple subcultures only on unconventional media (7H11 slant). PCR amplification was inconclusive. MICs for the isolate were determined using antifungal susceptibility testing performed on the isolate by an outside laboratory. The observed MICs are fluconazole (2.0), itraconazole (<=0.03), posaconazole (0.06) and voriconazole (0.06).

The characteristics of this isolate are somewhat atypical, and the isolate was not identified by conventional PCR; we conducted whole-genome sequencing using the Oxford Nanopore Technologies MinION platform. Sequence reads were massively aligned against the nonredundant GenBank database, with the vast majority of matches obtained against *B. gilchristii*. Additionally, more than 98% of the reads could be mapped unambiguously and with almost complete coverage of the *B. gilchristii* reference genome (strain SLH14081). The sequence reads were submitted to the NCBI SRA and are accessible through BioProject PRJNA767594.

### Disease prevalence in Tennessee

*Blastomyces* and *Histoplasma* infections are prevalent in Tennessee and are most commonly diagnosed in our laboratory by antigen detection tests offered through a commercial laboratory. The number of positive cases from the years 2020–2024 is shown in Table 1.

**Table 1.** Results from *Blastomyces*/*Histoplasma* antigen testing from clinical samples submitted to the Bacteriology and Mycology Laboratory, College of Veterinary Medicine, University of Tennessee. The test was sent out to a commercial laboratory (MiraVista Diagnostics, Indianapolis, IN) from suspect cases

	*Blastomyces*/*Histoplasma* antigen test results			
	Positive/total tested			
Year	Dogs	Cats	Other species	Positive all/total all (% positive)
2020	42/229	16/97	4/18	62/344 (18)
2021	33/249	9/103	10/17	52/369 (14)
2022	26/315	12/107	6/24	44/446 (9.9)
2023	44/367	24/118	7/26	75/511 (14.7)
2024	16/277	21/151	9/29	46/457 (10)
Positive/total (% positive)	161/1,437 (11.2)	82/576 (14.2)	36/114 (31.6)	279/2,127 (13.1)

## Discussion

Blastomycosis in humans and animals is usually caused by the dimorphic fungi *B. dermatitidis*, a pathogen with a wide geographic range in North America. The genus *Blastomyces* belongs to the family *Ajellomycetaceae* and the order Onygenales. The genus was initially believed to comprise only a single species, *B. dermatitidis*. However, multiple additional species have since been identified, including the cryptic species *B. gilchristii*. *B. gilchristii* has been isolated in Ontario, Wisconsin and Minnesota, mainly from human, canine and environmental samples [5]. The natural habitat of *B. dermatitidis* may be restricted to a particular microenvironment, and similarly, *B. gilchristii* may also have hyperendemic hotspots [6]. In addition, *B. gilchristii*, originally identified as *B. dermatitidis*, was reported to be highly virulent in immunocompetent humans and mice [5]. Blastomycosis has been previously documented in nondomestic cats in East Tennessee; however, infection with *B. gilchristii* in humans or animals has not yet been described in this part of the USA. *B. gilchristii* was found to be a genetically different monophyletic clade within *B. dermatitidis*. Other species within *Blastomyces* have been discovered, namely, *Blastomyces percursus* in the Middle East and Africa, *Blastomyces helicus* in the western USA and Canada and *B. emzantsi* in the Middle East and India, and they are all believed to be confined to particular geographic regions [3,4]. Cryptic species have also been found in other dimorphic pathogenic fungi such as *Histoplasma* and *Coccidioides* [7]. Although the ecological niche of blastomycosis is not well understood, previous studies have suggested that *B. gilchristii* is limited to the northern USA and Canada and is not detected in the southern USA [8]. Isolating this organism in the southeastern USA suggests that its geographic span is wider than previously suggested.

A recent report suggested that *B. gilchristii* is likely responsible for a high proportion of clinical blastomycosis cases in humans [6]. *B. gilchristii* has also been reported to be a cause of fatal acute respiratory syndrome in humans [9]. Genomic sequencing is an ideal strategy to identify case clusters caused by cryptic pathogens and may provide valuable insights into the epidemiology of these infections.

Cryptic fungal species, while morphologically indistinguishable from their known relatives, are genetically distinct and represent a significant yet often underappreciated cause of fungal diseases [5]. Cryptic fungal pathogens in multiple fungal genera have been reported, and the clinical impact of these pathogens is not well understood. Identifying a cryptic pathogenic fungal species as described in this report can be important to study changing patterns of prevalence and risk factors, and possible treatment options. Fungal diseases such as blastomycosis and histoplasmosis are prevalent in canine and feline species in our region, and these animals can also act as sentinels for various infectious diseases. Habitat and antifungal susceptibility patterns may be different for these undescribed species. It has been suggested that there are different virulence factors associated with the different species of *Blastomyces* in humans [10]. Application of whole-genome sequencing followed by phylogenetic and population genetic approaches is desirable to understand the evolutionary differences between isolates and their potential impact on pathogenesis. These approaches can be applied in public health settings for evaluating trends in prevalence, pathogen evolution and emergence. Assessing and documenting infection trends and environmental prevalence may provide valuable information on disease characteristics and incidence.
